# Shining the Spotlight on Multiple Daily Insulin Therapy: Real-World Evidence of the InPen Smart Insulin Pen

**DOI:** 10.1089/dia.2023.0365

**Published:** 2024-01-05

**Authors:** Janice MacLeod, Glen Heungyong Im, Madison Smith, Robert A. Vigersky

**Affiliations:** Medtronic Diabetes, Northridge, California, USA.

**Keywords:** Connected insulin pens, Continuous glucose monitoring, Precision insulin management, Smart insulin pen, Time in range, Time below range

## Abstract

**Objective:**

Connected insulin pens are creating opportunities for the millions of individuals with diabetes using multiple daily injections (MDI) therapy across the globe. Continuous glucose monitoring (CGM) data from connected insulin pens are revealing gaps and opportunities to significantly improve care for this population. In this article, we report real-world findings of the InPen™ smart insulin pen paired with CGM (InPen system), used by persons with type 1 diabetes (T1D) and type 2 diabetes (T2D).

**Methods:**

A retrospective cohort analysis was conducted with the real-world data collected from the InPen system of individuals (*N* = 3793 with T1D, *N* = 552 with T2D, and *N* = 808 unidentified) who used the system from January 01, 2020, to December 31, 2021. Diabetes management (e.g., missed and mistimed insulin dosing, mismatched food intake, and correction dose delivery) and glycemic outcomes were assessed.

**Results:**

In the overall and T1D populations, a dosing frequency of ≥3 doses per day and a missed dose frequency of <20% was associated with improved glycemia. In adults with T2D, missing <20% of doses was the significant factor determining improved glycemia.

**Conclusion:**

This analysis, integrating data from a smart insulin pen and CGM, provides insights into the impact of dosing behavior on glycemic outcomes and informs counseling strategies for the diabetes care team, through technologically advanced insulin management for those using MDI therapy.

## Introduction

The rapidly growing prevalence of diabetes is challenging health care systems, providers, and patients across the globe and forcing evaluation of cost-effective measures that support this complex, chronic, and largely self-managed condition. According to World Health Organization (WHO) estimates, there are currently 420 million people worldwide living with diabetes with about 9 million people with type 1 diabetes (T1D) relying on insulin for survival and ∼63 million individuals with type 2 diabetes (T2D) who require insulin for treatment.^1^ It is estimated that only two-thirds of individuals with T1D^2,3^ and <20% with insulin-treated T2D^3^ are achieving the American Diabetes Association (ADA) A1C target of <7.0%.

Since the discovery of insulin in 1921, multiple advancements in insulin development and delivery have been made, including ultrarapid and ultra-long-acting insulins, insulin pens, and most recently, the introduction of connected (i.e., tracking and smart) insulin pens and caps.^4,5^ While insulin pumps provide continuous insulin delivery for diabetes management, the rate of insulin pump use uptake varies considerably from country to country, and there are many barriers for individuals on insulin therapy that include access, cost, perceived complexity, and patient or provider preference.^6^ While use of continuous glucose monitoring (CGM) is growing, including among patients on injection therapy, a corresponding improvement in reaching glycemic targets has not been realized.^2,7^ Data from smart insulin pens and connected devices are shining the spotlight on multiple daily injections (MDI) therapy, revealing both gaps and opportunities to significantly improve care for this population.^8^

In this article, we report real-world data of the InPen™ paired with CGM (InPen system) and the impact of dosing behaviors on glycemia.

## The Roadmap to Smart Insulin Pens

Noting that “one size fits all” does not work when it comes to diabetes technologies, the ADA includes connected insulin pens in the Medical Standards of Care insulin therapy recommendations as a solution to potentially help patients on injection therapy with dose capture, dose recommendations, and dose titration.^9^ Similar recommendations are found in the American Association of Clinical Endocrinology Clinical Practice Guideline for the use of advanced diabetes technology.^10^ Given these standards and guidelines, there have been important steps toward providing advanced therapy to individuals choosing multiple daily insulin administration for diabetes management.

**FIG. 1.**
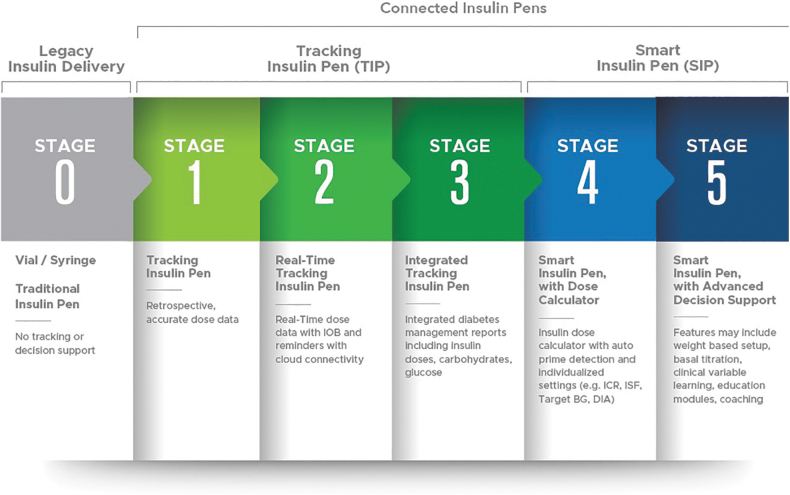
Connected insulin pen development (from Kerr and Warshaw^11^).

Kerr and Warshaw^11^ described five stages in connected insulin pen development (Fig. 1). Stage 0 includes legacy insulin delivery devices such as the vial and syringe and disposable insulin pens: offering no dosing support. Stages 1–3 are tracking insulin pens that provide retrospective dose tracking capability on an application (or app), cap, or clip. Stage 2 specifically involves devices that track insulin doses in real time through cloud connectivity, offering the ability to differentiate priming from therapy doses and objectively tracking active insulin, while providing missed dose alerts. Stage 3 devices integrate related data into diabetes management reports for visualization and sharing with the diabetes care team.

Stages 4 and 5 describe smart insulin pens, where Stage 4 devices add dose calculator functionality with individualized dose recommendations based on current glucose, meal data, programmed insulin therapy settings, and active insulin (or insulin on board). However, it is noted that not all currently available smart insulin pen technologies with dose calculator functionality have every feature listed within Stage 4 (e.g., dose amount tracking and auto prime detection). Stage 5 includes systems (currently in development) that provide advanced decision support based on real-time integrated CGM data analysis. These advances are projected to offer digital support to facilitate precision insulin management,^4^ previously available only to those on pump therapy, to individuals on insulin injection therapy.^12^

Research from individuals using MDI therapy with CGM and connected insulin pen or cap devices revealed that missed, mistimed, and mismatched insulin bolus doses are common and occur in both older and younger populations with T1D or T2D, and are associated with higher A1C.^13–18^ Numeracy challenges^19–21^ and the risk of insulin stacking^22^ add to the burden of daily insulin management for individuals on MDI therapy and the interventions that may be required from their care teams.

Connected insulin pens are now creating opportunities for clinicians to have a more complete picture of insulin dosing behavior.^23,24^ This visibility can potentially help address therapeutic inertia; the failure to initiate or intensify therapy in a timely manner according to evidence-based guidelines.^25^ Therapeutic inertia is of particular concern with insulin therapy, as both the ADA and the American Association of Clinical Endocrinology (AACE) have recommended the titration of insulin doses every 2 to 3 days or twice per week until glycemic targets are met with ongoing adjustments as needed, as insulin requirements change over time.^9,26^ In fact, a retrospective analysis of InPen system data indicated 60% of device users required adjustments in their basal insulin dose.^27^

There are many barriers to timely insulin titration and ongoing insulin management in clinical practice that include insufficient resources to adequately educate the patient, make frequent dose adjustments, or design and implement more complex insulin therapy regimens. The objective of the present study was to assess the real-world impact of dosing behaviors on diabetes management and glycemic outcomes of individuals using the InPen smart insulin pen system.

## Methods

### Study objective and population

This retrospective cohort analysis was conducted on data based on a randomly generated unique identifier that did not include personal identifiable user information (i.e., pseudonymization). Data were collected from adult and pediatric users of the InPen system (i.e., the InPen smart insulin pen [Medtronic, Northridge, CA] paired with a personal CGM system) who used the system for at least 30 days between January 2020 and December 2021. The analyzed data set included users' last 14 days of bolus dosing and CGM data. Users without at least one bolus record and <70% of sensor glucose (SG) data available in the last 14 days between the first 30 to 59 days of InPen use were excluded. The first 30 days were excluded from the analysis to avoid measuring dosing behaviors or glycemic outcomes associated with the onboarding of the diabetes technology.

The demographic information (age, diabetes duration, type of diabetes, and gender) was self-reported by the user into the InPen app and was optional. As the present study did not involve subject recruitment, enrollment, or participation in a trial and did not fall under human subject protection requirements (per the Department of Health and Human Services, Code of Federal Regulations, Title 28, Chapter 1, Part 46), Internal Review Board approval or exemption was not necessary.

### Methods to identify dose count and type

The InPen app tracks the timing and dosing amounts of rapid-acting insulin only. Long-acting insulin doses can be manually recorded by the user and reminders can be set for taking long-acting insulin doses. The rapid-acting insulin dose recommendations (from the app) are based on one of the dosing features that can be selected: carbohydrate counting; estimated small, medium, or large-sized meals; or fixed doses. The time and amount of each bolus was automatically captured and recorded by the InPen system.

Users also had the option to manually log doses taken with an insulin injector other than the InPen, within the app. They could also use the app-based dose calculator to calculate meal, meal+correction, correction-only doses, or dose without using the dose calculator. The dose count was measured as the average number of rapid-acting doses delivered or manually logged per day. Doses were categorized as correction-only when the dose amount delivered matched or was less than the amount that would have been recommended based on users' therapy settings. When the dose was 1 U or more than a calculated correction-only dose, it was categorized as a meal or meal+correction dose. The frequency of correction doses was then calculated as the average number of correction-only doses divided by all bolus doses delivered each day.

### Identification of missed and mistimed doses and missed correction opportunities

Bolus time was defined as the automatically recorded InPen system time or users' manually entered time. A rate-of-change detection methodology for SG data was used to detect meal events. At each detected meal event, the time and the timeliness of bolus injection were determined based on the time of the start of the glucose rise for each detected meal event. A bolus was deemed *on-time* when it was administered between 30 min before and ≤15 min after the start of the rise. A bolus was considered late when the injection time was between >15 min after and ≤60 min after the start of the rise. A missed dose was defined when the meal event was detected, but no record of bolus injection existed between ≤30 min before and ≥60 min after the start of the rise.

Each on-time, late, and missed dose counts were then divided by all detected meal events to generate the frequency (i.e., percentage). A *k*-means clustering analysis was used to identify three clusters of users based on bolus dose timing. Missed correction opportunities were defined as elevated SGs, in which the dose recommendations were no less than each users' correction threshold. Correction threshold was derived from the difference between 180 mg/dL and users' target glucose level, then divided by the insulin sensitivity factor.

### Statistical analysis

An ANOVA hypothesis test was used to assess significant difference in various average glycemic outcomes across groups, and a Tukey's post hoc analysis determined which groups differed when a difference was present, with significance level set to 0.05. The correlation between time-in-range (TIR) and on-time dose frequency was calculated using Pearson's *r*. All statistical analyses were conducted in Python™ software (Python Software Foundation, Fredericksburg, Virginia) using its SciPy.stats library. A *k*-means clustering was used to cluster users based on two variables: *on-time* dose and *missed* dose frequency. The initiation method was *k*-means++ with numerical features standardized and conducted on BigQuery Machine Learning in the Google Cloud Platform (Google, Mountain View, CA). Continuous variables are presented as mean ± standard deviation, while categorical variables are presented as *n* (%).

## Results

The demographics (age, duration of diabetes, diabetes type, and so on) of the overall study population (*N* = 5153) are listed in Table 1. Users were separated into six groups based on calculated average dose count per day. Table 2 shows the mean glycemic outcomes (time below range [TBR], TIR, time above range [TAR], and glycemic management indicator [GMI]), as well as the correction frequency, in each dose count group. Users tended to have lower TAR and GMI, as they dosed more often with a higher correction frequency. Groups had a significant difference in mean TIR (*P* < 0.05) based on the number of injections per day (Table 2).

**Table 1.** User Demographics and Characteristics

Characteristics	0–17 Years (*N* = 1090)	≥18 Years (*N* = 3368)	Age not specified (*N* = 695)
Age (years)	11.7 ± 4.2	43.4 ± 17.2	—
Duration of diabetes (years)	4.1 ± 5.9	16.8 ± 13.5	—
Weight (kg)	47.6 ± 21.0	80.8 ± 22.5	—
Diabetes type (*N*)			
Type 1 (*N* = 3793)	1003	2602	188
Type 2 (*N* = 552)	9	518	25
Type not specified (*N* = 808)	78	248	482
Gender (*N*)			
Male	468	1304	76
Female	513	1740	74
Gender not specified	109	324	545

Data are shown as mean ± SD or count.

SD, standard deviation.

**Table 2.** Glycemic Outcomes and Correction Frequency by Dose Count Group

Dose count group	*N*	TBR (%)	TIR (%)	TAR (%)	GMI	Correction frequency (%)
≥1	462	1.81	50.8	47.3	8.01	19.7
≥2	1447	1.66	52.3	46.0	7.84	15.7
≥3	1546	1.75	54.7	43.6	7.66	17.2
≥4	858	2.23	55.2	42.6	7.59	20.4
≥5	468	2.42	58.2	39.3	7.44	23.8
≥6+	382	2.51	61.0	36.4	7.33	24.1

TBR, time below range; TIR, time-in-range.

**FIG. 2.**
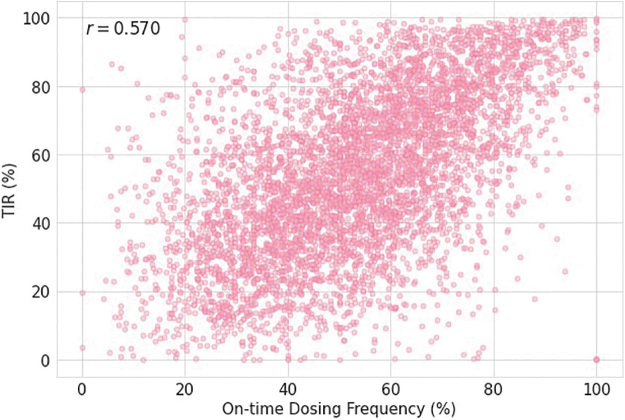
Correlation of on-time doses to TIR. TIR, time-in-range.

Cluster analysis demonstrated that Cluster 1 (*N* = 1125) had the lowest adherence to on-time dosing and the suboptimal glycemic outcomes, while Cluster 3 (*N* = 1889) demonstrated the highest adherence to on-time dosing and had better glycemic outcomes. Cluster 2 (*N* = 2139) had outcomes that fell between Clusters 1 and 3 (Table 3). All three groups had a significant difference in mean TIR (*P* < 0.0001) and TAR (*P* < 0.0001). The frequency of on-time dosing was significantly correlated with user TIR (*r* = 0.570, *P* < 0.0001, Tukey's test), as illustrated in Figure 2.

**Table 3.** Impact of On-Time, Missed, and Late Doses on Glycemia by Cluster

	Overall population	Cluster 1 (*N* = 1125)	Cluster 2 (*N* = 2139)	Cluster 3 (*N* = 1889)
Dose timing (%)				
On-time	53.3 ± 19.1	27.9 ± 8.5	49.4 ± 0.8	72.9 ± 1.0
Missed	34.9 ± 17.7	59.8 ± 9.2	37.2 ± 7.2	17.3 ± 7.8
Late	11.8 ± 8.1	12.3 ± 7.3	13.3 ± 12.3	9.9 ± 7.6
Glycemic outcomes (%)				
TBR	1.9 ± 3.7	1.6 ± 2.8	1.7 ± 2.6	2.4 ± 5.0
TIR	54.6 ± 23.6	37.4 ± 20.5	50.9 ± 20.5	69.0 ± 19.8
TAR	43.5 ± 24.2	61.0 ± 21.1	47.5 ± 21.1	28.6 ± 20.3
Age (*N*)				
0–17	1090	291	554	245
18–64	2935	643	1136	1156
65 or older	433	50	162	221
Not specified	695	141	287	267
Diabetes type (*N*)				
Type 1	3793	833	1601	1359
Type 2	552	97	219	236
Not specified	808	195	319	294

Data are shown as mean ± SD or count.

The average duration of missed correction opportunities per day showed strong negative correlation with TIR (*r* = −0.852, *P* < 0.0001) (Supplementary Fig. S1). For dosing behavior that included two or less versus three or more doses per day and a missed dose frequency above and below 20%, the impact on glycemia is presented for the overall population (Table 4), adult T1D users (Supplementary Table S1), pediatric T1D users (Supplementary Table S2) and adult T2D users (Supplementary Table S3).

**Table 4.** Dosing Behavior Impact on Overall Glycemia

	Dose rate <3 doses/day (*N* = 1909)	Dose rate ≥3 doses/day (*N* = 3244)	
	—	Missed dose rate ≥20% (*N* = 2282)	Missed dose rate <20% (*N* = 962)
Dose timing (%)			
On-time	44.5 ± 19.7	51.3 ± 13.2	75.4 ± 11.1
Missed	46.7 ± 17.4	34.6 ± 10.3	12.0 ± 5.4
Late	8.8 ± 6.7	14.1 ± 8.1	12.6 ± 8.8
Correction dose (%)	16.7 ± 21.4	21.5 ± 19.3	15.9 ± 14.9
No. of detected meals	3.9 ± 1.2	5.0 ± 1.2	4.6 ± 1.3
Glycemic outcomes (%)			
TBR	1.7 ± 3.6	1.7 ± 2.6	2.9 ± 5.6
TIR	52.0 ± 26.0	50.1 ± 20.2	70.3 ± 19.2
TAR	46.3 ± 26.4	48.2 ± 20.8	26.9 ± 19.8
GMI	7.9 ± 1.3	7.8 ± 0.9	7.0 ± 0.8
Age (*N*)			
0–17	401	601	88
18–64	1115	1191	629
65 or older	163	172	98
Not specified	230	318	147
Diabetes type (*N*)			
Type 1	897	1807	734
Type 2	229	120	80
Not specified	783	355	148

Data are shown as mean ± SD or count.

In the overall and T1D populations, a dosing frequency of 3+ doses/day and a missed dose frequency of <20% were associated with better glycemic outcomes compared to those with a lower dosing frequency or higher missed dose rate. There were similar outcomes in adults with T1D, children with T1D and adults with T2D. Manually tracked basal dose amount data were available for 67.2% of the users in this analysis. The average basal/bolus split for users with basal data was 50.7 U/49.3 U.

## Discussion

While CGM has become the standard of care for those on MDI therapy, patients are still not achieving glycemic targets in this population. This large-scale real-world analysis of integrated data from the InPen system provides unique insights and visibility into the dosing behaviors and their impact on glycemic outcomes in patients on MDI therapy and adds to the body of literature on connected insulin delivery devices paired with CGM.

Missed and mistimed insulin doses are common in MDI therapy as verified in this analysis of smart insulin pen users. Research from individuals using MDI therapy with CGM devices and connected insulin pen or cap devices shows that late or missed insulin boluses are associated with higher A1C.^16,18^ In a prospective, observational trial of 94 participants with T1D, the use of a connected insulin pen with CGM resulted in significant improvements in glycemia and a significant decrease in missed doses.^13^ In a systematic review, 20%–45% of participants on insulin therapy reported the mistiming of their insulin doses^17^ citing disrupted daily routines, social situations, and hypoglycemia avoidance as reasons.

It is estimated that two out of every three adults in the United States are unable to perform basic math due to limited numeracy skills.^20^ Numeracy challenges in people with diabetes have the potential to impact the ability to calculate insulin doses and achieve glycemic targets.^22,23^ Insulin stacking (overlap of bolus doses) is estimated to occur in 60% of insulin bolus doses putting the individual at risk for hypoglycemia.^22^ In an observational study of 50 participants with T1D on MDI therapy using a Bluetooth-enabled pen cap and CGM, it was noted that 37% of total boluses resulted in persistent 3-h postprandial hyperglycemia, while 10% resulted in clinically significant 3-h postprandial hypoglycemia (glucose <55 mg/dL).^18^

A study of pediatric individuals with T1D identified six habits associated with reduction of HbA1c.^28^ Four of these six habits are related to insulin delivery, demonstrating the importance of ensuring that individuals with diabetes have the support they need to manage the complexity of determining when to deliver an insulin dose and how much insulin is needed. With use of CGM and standard insulin injection therapy, patients may struggle with maintaining the aforementioned insulin delivery-related habits. With the use of an insulin pump or smart insulin pen, however, they may be able to close the gap in support they experience while managing a complicated insulin regimen.^28^

Smart insulin pens support individuals on MDI therapy with intelligent algorithms and features (e.g., objective active insulin tracking, differentiating priming from therapy doses, and dose recommendations) allowing opportunities to safely deliver more frequent rapid-acting insulin doses, including correction doses. This analysis, in fact, shows that increased correction dose frequency is associated with improved TIR.

A strength of this study is that it is the first exploration and analysis of behaviors and outcomes for MDI patients using real-world data. The ability to achieve this relates to the InPen system's capability of tracking both dosing behaviors and CGM trends. The importance of using real-world data cannot be overemphasized. While clinical trials are critical to understanding the effectiveness of medical devices, they are often conducted with a small number of subjects. Real-world data not only permit the analysis of a large data set that includes both sensor glucose and the time and amount of the insulin dose but also importantly reflect the actual conditions that patients encounter in daily life. These conditions generally include nonalgorithmic insulin dosing, missed or late meal bolusing, and inaccurate carbohydrate counting.

The current study has some limitations. First, the population for the analysis only included those who used compatible real-time CGM along with the smart insulin pen. While it is CGM that enabled measuring glycemic management metrics reported herein, a follow-up study based on blood glucose monitoring would be required to provide evidence of benefit for those using a smart insulin pen without CGM. Second, the study period is limited to 30 days, not during the entire duration of the usage. A study with longer duration would supplement the findings around the benefits of smart insulin pen use. Demographic information on race/ethnicity was not available in this real-world analysis.

Another constraint lies in the approach of the analyses taken in this study. Based on the real-world data, the analyses were designed to compare between groups split from the population. This may be useful to reveal the difference between the groups by the certain behaviors, which was an aim for this study. However, it does not prove the direct causal relationship between variables. For example, one of the analyses revealed that those who deliver bolus insulin more often showed higher TIR. While it is tempting to conclude that the more one doses, the better the outcome, other factors such as how often doses are delivered on time, late, or missed, along with whether users have optimized therapy settings, would also contribute toward the expected outcome.

On an individual basis, data derived from smart insulin pens may be used to inform counseling strategies for the diabetes care team and prioritize educational and behavioral diabetes management goals for those on MDI therapy. Intelligent dosing technology can support both the person on MDI therapy and their care team, further reducing the considerable burden of insulin delivery. By combining dosing data with CGM data, it will be possible to provide glucose alerts only when action is needed (e.g., when a correction dose would be safe to administer based on high glucose and the amount of “insulin-on-board”). This may potentially reduce alarm fatigue and better guide individuals in daily decision-making.

Meal detection technology could alert users after a meal to dose their insulin if a dose were omitted. In the future, artificial intelligence/machine learning can be applied to provide personalized long-acting and rapid-acting dosing recommendations that fine-tune insulin therapy settings, further reducing the burden of diabetes management.

## Conclusions

In summary, this real-world analysis of integrated smart insulin pen and CGM data provides insights into the impact of dosing behavior on glycemic outcomes. It demonstrates that smart insulin pen systems support MDI therapy patients in daily dosing decisions and may facilitate communication with the diabetes care team, which provides precision insulin management for all individuals on insulin therapy regardless of delivery method.

## Supplementary Material

Supplemental data

Supplemental data

Supplemental data

Supplemental data
