# Supplementary material for: Shining the Spotlight on Multiple Daily Insulin Therapy: Real-World Evidence of the InPen Smart Insulin Pen
Source: Diabetes Technol Ther. 2024 Jan 5;26(1):33–9. doi: 10.1089/dia.2023.0365 (PMC10794824; doi:10.1089/dia.2023.0365)

**Supplemental Materials**

**Supplemental Figure 1.** Correlation of Duration of Missed Correction Opportunities to TIR


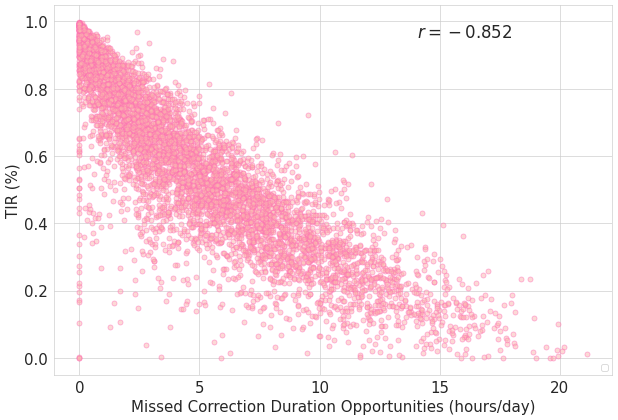

Supplement: Supplemental data [file Supp_FigS1.docx]
