# Supplementary material for: Shining the Spotlight on Multiple Daily Insulin Therapy: Real-World Evidence of the InPen Smart Insulin Pen
Source: Diabetes Technol Ther. 2024 Jan 5;26(1):33–9. doi: 10.1089/dia.2023.0365 (PMC10794824; doi:10.1089/dia.2023.0365)
Supplement: Supplemental data [file Supp_TableS1.docx]

**Supplemental Table 1.** Dosing Behavior Impact on Type 1 Glycemia (Adults)

|  | **Dose Count**  **< 2 doses/day**  **(N = 850)** | **Dose Count**  **≥ 3 doses/day**  **(N = 1,752)** | |
| --- | --- | --- | --- |
|  | **-** | **Missed Dose Rate**  **≥ 20%**  **(N = 1,153)** | **Missed Dose Rate**  **< 20%**  **(N = 599)** |
| **Dose timing (%)** |  |  |  |
| On-time | 42.5 ± 20.4 | 51.2 ± 13.3 | 75.2 ± 10.9 |
| Missed | 47.7 ± 17.9 | 33.9 ± 10.3 | 11.8 ± 5.4 |
| Late | 9.8 ± 6.9 | 14.9 ± 8.2 | 13.1 ± 8.5 |
| **Correction dose** **(%)** | 19.4 ± 22.5 | 22.5 ± 19.7 | 16.7 ± 14.9 |
| **Number of detected meals** | 3.9 ± 1.1 | 4.8 ± 1.1 | 4.7 ± 1.3 |
| **Glycemic outcomes (%)** |  |  |  |
| TBR | 2.1 ± 3.5 | 1.9 ± 3.0 | 2.9 ± 3.5 |
| TIR | 49.7 ± 25.7 | 50.2 ± 19.2 | 70.5 ± 18.6 |
| TAR | 48.3 ± 26.3 | 47.9 ± 20.0 | 26.7 ± 19.5 |
| GMI | 8.0 ± 1.3 | 7.8 ± 0.9 | 6.9 ± 0.7 |
| **Age** **(N)** |  |  |  |
| 18 – 64 | 785 | 1,044 | 540 |
| 65 or older | 65 | 109 | 59 |

Data are shown as mean ± SD or count.
