# Supplementary material for: Shining the Spotlight on Multiple Daily Insulin Therapy: Real-World Evidence of the InPen Smart Insulin Pen
Source: Diabetes Technol Ther. 2024 Jan 5;26(1):33–9. doi: 10.1089/dia.2023.0365 (PMC10794824; doi:10.1089/dia.2023.0365)
Supplement: Supplemental data [file Supp_TableS2.docx]

**Supplemental Table 2**. Dosing Behavior Impact on Type 1 Glycemia (Pediatric)

|  | **Dose Count**  **< 2 doses/day**  **(N = 357)** | **Dose Count**  **≥ 3 doses/day**  **(N = 646)** | |
| --- | --- | --- | --- |
|  | **-** | **Missed Dose Rate**  **≥ 20%**  **(N = 565)** | **Missed Dose Rate**  **< 20%**  **(N = 81)** |
| **Dose timing (%)** |  |  |  |
| On-time | 42.0 ± 16.8 | 50.2 ± 13.3 | 73.6 ± 11.7 |
| Missed | 49.6 ± 14.6 | 36.7 ± 10.4 | 13.3 ± 4.9 |
| Late | 8.4 ± 6.2 | 13.2 ± 7.8 | 13.1 ± 1.0 |
| **Correction dose** **(%)** | 16.3 ± 21.2 | 21.0 ± 18.6 | 15.2 ± 14.4 |
| **Number of detected meals** | 4.3 ± 1.2 | 5.3 ± 1.1 | 4.8 ± 1.2 |
| **Glycemic outcomes (%)** |  |  |  |
| TBR | 1.5 ± 2.0 | 1.6 ± 2.2 | 2.1 ± 2.8 |
| TIR | 51.4 ± 26.8 | 48.6 ± 21.6 | 66.8 ± 22.2 |
| TAR | 47.0 ± 27.0 | 49.8 ± 22.3 | 31.1 ± 22.9 |
| GMI | 8.0 ± 1.3 | 8.0 ± 1.0 | 7.1 ± 0.9 |
| **Age (N)** |  |  |  |
| 0 – 17 | 357 | 565 | 81 |

Data are shown as mean ± SD or count.
