# Supplementary material for: Shining the Spotlight on Multiple Daily Insulin Therapy: Real-World Evidence of the InPen Smart Insulin Pen
Source: Diabetes Technol Ther. 2024 Jan 5;26(1):33–9. doi: 10.1089/dia.2023.0365 (PMC10794824; doi:10.1089/dia.2023.0365)
Supplement: Supplemental data [file Supp_TableS3.docx]

**Supplemental Table 3**. Dosing Behavior Impact on Type 2 Glycemia (Adults)

|  | **Missed Dose Rate**  **≥ 20%**  **(N = 406)** | **Missed Dose Rate**  **< 20%**  **(N = 112)** |
| --- | --- | --- |
| **Dose timing (%)** |  |  |
| On-time | 51.6 ± 15.9 | 81.4 ± 9.1 |
| Missed | 41.0 ± 14.6 | 12.6 ± 5.5 |
| Late | 7.5 ± 5.9 | 5.9 ± 6.1 |
| **Correction dose** **(%)** | 11.4 ± 18.0 | 8.1 ± 12.9 |
| **Number of detected meals** | 3.9 ± 1.1 | 3.5 ± 1.0 |
| **Glycemic outcomes (%)** |  |  |
| TBR | 0.7 ± 1.6 | 0.7 ± 1.2 |
| TIR | 56.9 ± 23.6 | 75.9 ± 20.8 |
| TAR | 42.4 ± 23.9 | 23.4 ± 21.0 |
| GMI | 7.6 ± 1.0 | 7.0 ± 0.7 |
| **Age (N)** |  |  |
| 18 – 64 | 280 | 72 |
| 65 or older | 126 | 40 |

Data are shown as mean ± SD or count.
